# Healthy food marketing and purchases of fruits and vegetables in large grocery stores

**DOI:** 10.1016/j.pmedr.2019.100861

**Published:** 2019-03-30

**Authors:** Katherine Sutton, Julia Caldwell, Sallie Yoshida, Jack Thompson, Tony Kuo

**Affiliations:** a Division of Chronic Disease and Injury Prevention, Los Angeles County Department of Public Health, Los Angeles, CA 90010, USA; b Sarah Samuels Center for Public Health Research & Evaluation, Oakland, CA 94612, USA; c Department of Epidemiology, UCLA Fielding School of Public Health, Los Angeles, CA 90095, USA; d David Geffen School of Medicine at UCLA, Los Angeles, CA 90024, USA; e Population Health Program, UCLA Clinical and Translational Science Institute, Los Angeles, CA 90095, USA

**Keywords:** Promotion strategies, Food marketing, Grocery stores, Fruits and vegetables

## Abstract

Healthy food marketing in the retail environment can be an important driver of fruit and vegetable purchases. In Los Angeles County, the *Nutrition Education and Obesity Prevention* (NEOP) program utilized this strategy to promote healthy eating among low-income families that shop at large retail chain stores. The present study assessed whether self-reported exposure to large retail NEOP interventions, including seeing at least one store visual, watching an in-store cooking demonstration, and/or seeing at least one program advertisement, were associated with increased fruit and vegetable purchases. During fall 2014, the Division of Chronic Disease and Injury Prevention in the Los Angeles County Department of Public Health partnered with Samuels Center to conduct store patron intercept surveys at six large food retail stores participating in NEOP across Los Angeles County. Of 1050 participants who completed the survey, almost a quarter (25.0%) reported seeing at least one visual throughout the store and 9.2% watched a cooking demonstration. Seeing at least one visual and watching a cooking demonstration were not significantly associated with percent dollars spent on fruits and vegetables each week. Among participants who reported being exposed to at least one store visual, those enrolled in the Supplemental Nutrition Assistance Program (SNAP) reported spending 6% more on fruits and vegetables than those who were not enrolled (*p* = 0.046). Although the NEOP store interventions did not *individually* increase store purchases, their educational value may still influence patron food selection, especially if coupled to the monetary resources of SNAP for those who are enrolled.

## Highlights

• Seeing a store visual was not associated with percent dollars spent on produce.
• Of those who saw a visual, females spent more on fruits and vegetables than males.
• SNAP enrollees spent 6% more than non-enrollees on produce.

## 1 Introduction

Prior research suggests that low-income individuals are less likely to consume the recommended amount of fruits and vegetables as compared to higher income individuals (Kirkpatrick et al., 2012). For example, in Los Angeles County, only 12% of those living at or below 200% of the Federal Poverty Level (FPL) are consuming 5 fruits and vegetables per day, as compared to 19% for those who were living at or above 300% FPL (Los Angeles County Department of Public Health. Office of Health Assessment and Epidemiology, 2015). The Supplemental Nutrition Assistance Program Education (SNAP-Ed)^2^ Project, locally known in Los Angeles County as the *Nutrition Education and Obesity Prevention* (NEOP)^3^ program, provided nutrition education and obesity prevention strategies that were consistent with the *Dietary Guidelines for Americans* to improve the nutrition and health of low-income participants.

Supermarkets or large retail grocery stores represent an optimal setting for interventions that seek to improve food purchase decisions. Point-of-purchase (POP)^4^ interventions usually include the use of printed materials such as signs and labels, food demonstrations, and taste-testing to draw attention to healthier food products or options (Glanz and Yaroch, 2004). Previous studies indicate that POP interventions are viable in low-income communities and can reach many individuals at a relatively low cost (Kristal et al., 1997; Sutherland et al., 2010; Lang et al., 2000; O'Loughlin et al., 1996). Past research of POP interventions in supermarkets have also found that these efforts are only modestly effective in a few cases and are generally not uniformly impactful for increasing targeted food purchases (Gittelsohn, 2010; Kristal et al., 1997; Dougherty et al., 1990). For example, Ogawa et al. (2011) found that using health-related posters for 3 months increased sales of total vegetables. Milliron et al.'s (2012) use of POP interventions resulted in greater purchasing of fruits and dark-green/yellow vegetables, but they required individual nutrition counseling.

The purpose of the present study was to assess whether healthy purchases increased after six large retail grocery stores in Los Angeles County participated in the NEOP Project's store marketing interventions. In particular, the study examined whether self-reported exposure to the in-store marketing interventions, including seeing at least one store visual, watching an in-store cooking demonstration, and/or seeing at least one NEOP mass media advertisement in the community, were associated with increased fruit and vegetable purchasing. A secondary aim of the study was to assess, among those who reported seeing at least one store visual, whether fruit and vegetable purchases differed by selected subgroups. To the best of our knowledge, the present study is the first of its kind to examine grocery store POP interventions as part of a broader obesity prevention initiative in Los Angeles County.

## 2 Methods

### 2.1 The nutrition education and obesity prevention healthy retail food program in Los Angeles County

**Fig. 1:**
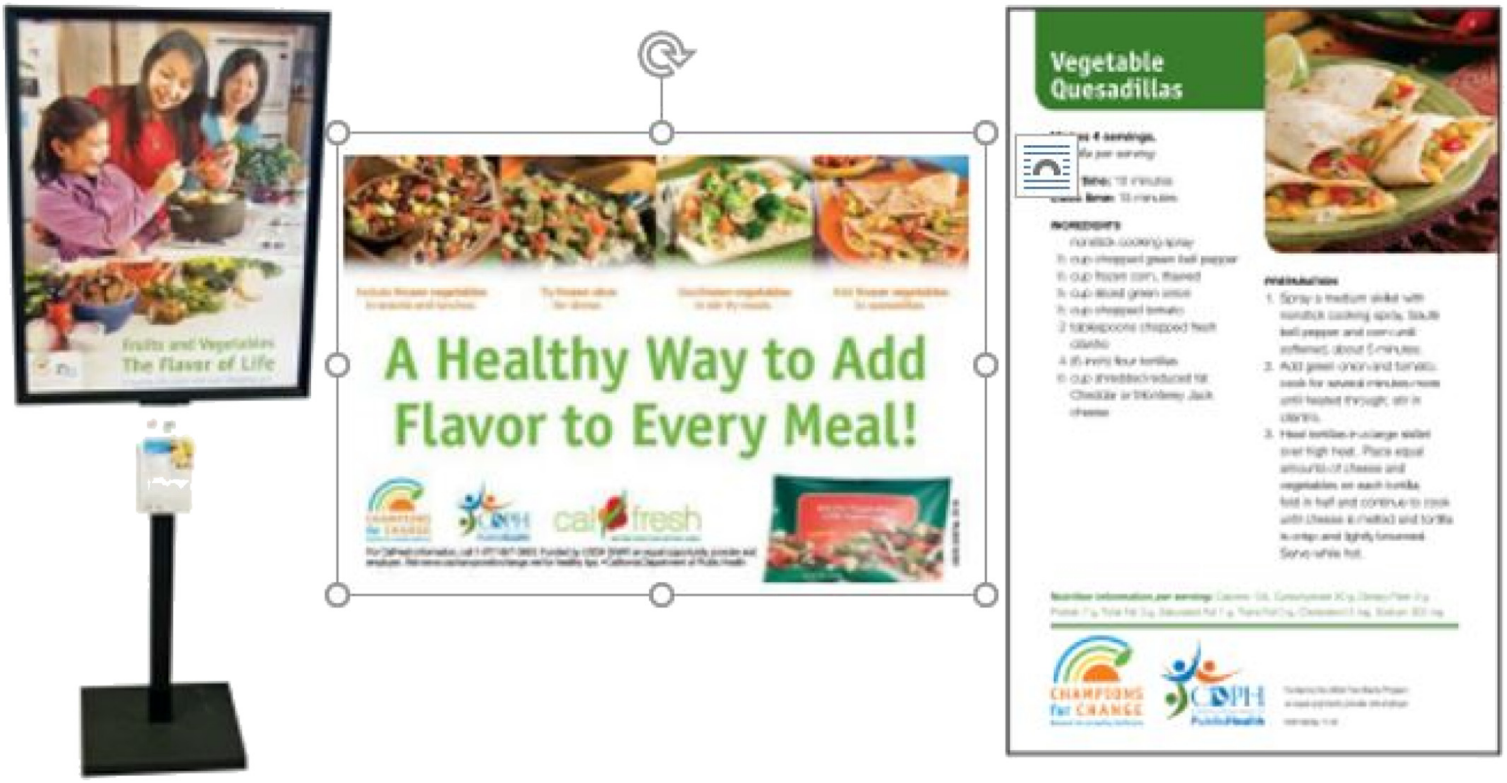
Examples of marketing materials posted throughout a store.

The Los Angeles County Department of Public Health's (DPH)^5^ NEOP Retail Program, funded through SNAP-Ed dollars, encouraged low-income individuals to make healthier purchasing choices at grocery stores by providing POP marketing materials and by conducting food demonstrations to highlight the benefits of consuming fruits, vegetables, and other healthy food and beverages. The program used freezer clings, recipe cards, shelf wobblers, and floor stands as marketing materials throughout the store to promote healthy food purchases (see examples in Fig. 1). Food demonstrations and test-tasting involving fruits and vegetables were also provided at the store to expose customers/patrons to unfamiliar produce and give them ideas on preparing these foods at home. Designed to improve public awareness of healthy eating, a NEOP mass media campaign (*Champions for Change*) was implemented in the field concurrently to the Retail Program. This campaign included billboards, radio, public transit, and commercial advertisements that promoted fruit and vegetable consumption and healthy living.

### 2.2 Study design

A cross-sectional intercept survey of patrons at six large retail grocery stores that participated in the local NEOP Retail Program was conducted to assess fruit and vegetable purchasing behaviors and exposure to the NEOP intervention signage and/or educational messaging/intervention throughout the store. Data collection took place between October 2014 and February 2015 at the six large food retail stores. These stores were in SNAP eligible census tracts (i.e., 50% or more of the population in the geographic area must be at or below 185% of the FPL), consisting of downtown Los Angeles, South Los Angeles, and East Los Angeles neighborhoods. At each store location, four trained staff systematically approached prospective participants as they left the store after shopping. A concurrent series of environmental assessments were also conducted at each of the six stores.

### 2.3 Study population and analytic samples

The study population comprised of adult patrons from the six retail grocery store locations. To be eligible for the survey, they must have been at least 18 years of age, speak English or Spanish, be willing and able to complete the administered survey, and be a store patron exiting the grocery store. An incentive in the form of a hat and/or t-shirt was given to all participants who completed the survey partially or in its entirety.

A 23-item street-intercept survey instrument was verbally administered to each participant using QuickTapSurvey (QuickTapSurvey, 2019). Each survey took approximately 10–15 min to complete. The survey instrument collected demographic information, food purchasing characteristics of store patrons, perceptions of the store environment, and information about exposure to the NEOP Retail interventions as they relate to the dollar amount spent on fruits and vegetables.

A total of 1101 individuals were administered the survey. Thirty-five of them who did not answer the question on seeing a visual in the store and 16 others whose reported spending on produce exceeded the total amount they spent on groceries each week (i.e., fruits/vegetables >100% of their total purchase) were excluded. For the multivariable regression analysis, participants with missing values on any of the study variables were also excluded. In total, two analytic samples were used in the modeling analyses: (1) all participants who successfully enrolled in the survey and did not have missing values in their responses (*n* = 929) and (2) a sub-sample of participants who saw at least one visual in the store (*n* = 236).

### 2.4 Measures

#### 2.4.1 Fruit and vegetable purchasing behavior

The outcome variable was derived from three questions. Participants were asked (1) ‘on average, how much do you spend on groceries each week?’ and of that amount how much is spent on (2) fruits and (3) vegetables. The percent of dollars spent on produce was calculated by dividing the dollar amount spent on fruits and vegetables by the total dollar amount spent on groceries.

#### 2.4.2 NEOP intervention exposure

The main exposure of interest was whether participants (adults) observed a visual in the store while they shopped. During the survey, participants were shown five possible visuals and asked: ‘Which of the following have you seen in this store?’ (A, B, C, D, E, none of the ads). Participants were categorized as exposed if they reported observing at least one store visual (yes/no). Participants were also asked about the exposure to cooking demonstrations: ‘Have you seen any *Champions for Change* healthy food cooking demonstrations?’ and if so, ‘Did you stay to watch the demonstration?’ These participants were categorized as exposed if they stayed to watch the demonstration (yes/no). Lastly, participants were asked about seeing any NEOP (SNAP-Ed/CalFresh) media ads in the community: ‘Have you seen any of these [campaign was called *Champions for Change*] ads’ (A, B, C, D, E, F?). Participants were categorized as seeing a community level visual if they reported seeing at least one of these visuals (yes/no).

#### 2.4.3 Socio-demographic characteristics

Covariates used in the present study analyses included gender (female/male), age (18–35, 36–50, 51–64, ≥65), ethnicity (Latino/non-Latino), educational attainment (less than high school, high school graduate or GED, associate degree, Bachelor's degree and higher), and employment status (currently working/currently not working).

Participants were asked whether anyone in their household receives CalFresh benefits (i.e., Food Stamps, Supplemental Nutrition Assistance Program, Electronic Benefits Transfer) (yes/no). Participants were asked whether their household receives Special Supplemental Nutrition Program for Women, Infants, and Children (WIC) (yes/no) or Head Start (yes/no). The analyses controlled for store locations (i.e., three stores in downtown Los Angeles, one store in East Los Angeles, and two stores in South Los Angeles). The analyses also controlled for accurate knowledge of U.S. Department of Agriculture's *My Plate* recommendations, since individuals who are more knowledgeable about fruit and vegetable consumption may purchase a higher proportion of produce. Participants were asked: ‘If we were to put all of our dinner food on a one plate, how much of it do you think should be fruits and vegetables?’ Would it be: (i) ¼; (ii) 1/3; (iii) ½; or (iv) ¾. Those reporting ‘½’ were categorized as correct (1) and all other responses were categorized as incorrect (0).

#### 2.4.4 Environmental assessments

Environmental assessments were conducted by trained staff at all six store locations. The assessment tool and related protocols were adapted from the Grocery Marketing Environmental Assessment (GMEA) (Kerr et al., 2012). The tool assessed the marketing environments of the checkout aisles, produce section, and main soda aisle. The tool documented the presence of fresh fruits and vegetables in five ‘High Impact Areas,’ i.e., places throughout the store that have been shown to have a higher than average effect on purchasing patterns: the store entrance, the endcaps at the end of each aisle, islands throughout the store, and the checkout aisles. The tool also evaluated healthy food and beverage messaging throughout the store.

### 2.5 Data analysis

Descriptive statistics were generated for the survey participants' demographic characteristics. For the analytic samples, ordinary least squares regression (OLS) was used to test the relationships between NEOP intervention exposures and fruit and vegetable purchasing for all participants, adjusting for socio-demographic characteristics, public assistance enrollment, store location, and fruit and vegetable knowledge. Since the main intervention strategy for the large retail grocery stores were the marketing materials, a more focused analysis assessed whether the association between store visual exposure and fruit and vegetable purchasing varied by selected sub-group. For the latter analysis, a more restricted sample of participants who reported seeing any visual in the store was used. All study analyses were conducted in SAS v.9.4 (SAS Institute, Inc., Cary, North Carolina). The Institutional Review Board at DPH reviewed and approved this project prior to data collection.

## 3 Results

### 3.1 Intercept survey findings

Participant characteristics for the entire sample (*n* = 1050) are summarized in Table 1. A majority of participants were Latino (91.2%), female (72.2%), and high school graduates or less (88.3%). Of the store patrons (shoppers), 31.6% received SNAP benefits and 23.4% received WIC. Almost a quarter (25.0%) reported seeing at least one visual throughout the store and 9.2% watched a cooking demonstration. On average, participants reported spending a total of $118.19 on groceries and $48.80 on fruits and vegetables each week (43%).

**Table 1:** Demographic characteristics of intercept survey participants, Los Angeles County, 2014–2015.

	Total	
	*n*	% or Mean (SD)
Dollars spent on groceries each week	1050	118.19 (75.1)
Dollars spent on fruits and vegetables each week	1050	48.80 (36.2)
Percent of dollars spent on fruits and vegetables	1050	43.03 (17.4)
Reported seeing one visual or more in store^A^	262	25.0
Watched cooking demonstration in store	97	9.2
Reported seeing at least one media ad in community^B^	603	57.6
Gender		
Female	747	72.2
Male	288	27.8
Age		
18–35	247	23.7
36–50	403	38.7
51–64	260	25.0
65+	132	12.7
Latino	906	91.1
Educational attainment		
Less than high school	458	44.4
High school	453	43.9
Associates	51	4.9
Bachelors +	70	6.8
Currently working	509	48.5
Enrolled SNAP/CalFresh	330	31.6
Enrolled WIC	244	23.4
Enrolled head start	42	4.0
Location		
Downtown Store 1	198	18.9
Downtown Store 2^C^	81	7.7
Downtown Store 3	202	19.2
East Los Angeles Store 4	196	18.7
South Los Angeles Store 5	170	16.2
South Los Angeles Store 6	203	19.3
Knowledge of fruit and vegetable recommendation^A^		
Correct	408	39.8
Incorrect	616	60.2

A Of a possible 5 store visuals.

B Of a possible 6 community visuals.

C Data collection had to be stopped due to neighborhood safety reasons.

Table 2 shows the relationships between report of exposure to the large retail intervention and percent of dollars spent on fruits and vegetables each week. After adjusting for socio-demographic characteristics, public assistance enrollment, store location, and fruit and vegetable knowledge, seeing at least one visual in the store and watching a cooking demonstration were not significantly associated with percent of total dollars spent on fruits and vegetables each week (Model 2). Seeing at least one (NEOP's *Champions for Change*) media advertisement in the community was not significantly associated with spending more on fruits and vegetables. Two socio-demographic characteristics were positively associated with fruit and vegetable purchases. Female participants reported spending 3% more on fruits and vegetables as compared to male participants (β = 0.03, 95% Confidence Interval [CI]: 0.01, 0.06, *p* = 0.019). Among participants with higher education (Bachelor's degree or more), they reported spending 6% more on fruits and vegetables as compared to those with less than a high school degree (β = 0.06, 95% CI: 0.01, 0.11, *p* = 0.016).

**Table 2:** Ordinary least squares regression analysis of large retail store exposure to NEOP interventions and percent of total dollars spent on fruits and vegetables among all survey participants, Los Angeles County, 2014–2015.

	Model 1 (bivariate)			Model 2 (multivariable)		
	β	95% CI	*p*-Value	β	95% CI	*p*-Value
Reported seeing at least one visual in store (ref = no)	0.01	−0.02, 0.03	0.580	0.01	−0.02, 0.04	0.554
Watched cooking demonstration in store (ref = no)	0.01	−0.02, 0.05	0.515	0.01	−0.04, 0.05	0.814
Reported seeing at least one Champions advertisement in community (ref = no)	0.02	0.00, 0.05	0.021	0.02	−0.01, 0.04	0.215
Female (ref = Male)	0.03	0.01, 0.06	0.007	0.03	0.01, 0.06	0.019
Age (ref = 18–35)						
36–50	−0.02	−0.04, 0.01	0.274	−0.02	−0.05, 0.01	0.210
51–64	−0.00	−0.03, 0.03	0.829	−0.02	−0.05, 0.02	0.401
65+	0.02	−0.01, 0.06	0.205	0.01	−0.03, 0.06	0.604
Latino (ref = non-Latino)	0.02	−0.02, 0.05	0.406	0.01	−0.03, 0.06	0.552
Educational attainment (ref ≤ high school)						
High school	−0.02	−0.04, 0.01	0.164	−0.01	−0.04, 0.01	0.296
Associates	−0.01	−0.06, 0.04	0.690	−0.02	−0.08, 0.04	0.475
Bachelors +	0.05	0.00, 0.09	0.033	0.06	0.01, 0.11	0.016
Currently working (ref = no)	0.00	−0.02, 0.02	0.919	0.01	−0.02, 0.03	0.597
Receives SNAP/CalFresh (ref = no)	−0.01	−0.03, 0.01	0.345	0.00	−0.03, 0.03	0.931
Receives WIC (ref = no)	0.00	−0.03, 0.02	0.980	−0.01	−0.04, 0.02	0.552
Receives head start (ref = no)	0.02	−0.04, 0.07	0.571	0.01	−0.05, 0.07	0.818
Neighborhood location (ref = Downtown Store 1)						
Downtown Store 2	0.04	−0.00, 0.09	0.0516	0.03	−0.02, 0.07	0.264
Downtown Store 3	−0.02	−0.05, 0.01	0.2496	−0.01	−0.05, 0.02	0.516
East Los Angeles Store 4	0.01	−0.02, 0.05	0.4929	0.01	−0.03, 0.05	0.622
South Los Angeles Store 5	0.03	−0.01, 0.06	0.1066	0.00	−0.04, 0.04	0.897
South Los Angeles Store 6	−0.01	−0.04, 0.02	0.6058	−0.02	−0.01, 0.02	0.348
Knowledge of fruit and vegetable recommendation (ref = incorrect)	0.02	−0.01, 0.04	0.147	0.00	−0.02, 0.03	0.917

Since one of the main intervention strategies was the introduction of the promotional visuals in the store, a restricted sample was used for the models in Table 3; this sample comprised of only participants who reported being exposed to at least one store visual. Among participants who saw at least one visual, females reported spending significantly more on fruits and vegetables as compared to males (β = 0.07, 95% CI = 0.01, 0.13, *p* = 0.029) (Model 2). Participants who were enrolled in in SNAP/CalFresh reported spending 6% more on fruits and vegetables as compared to those who were not enrolled in this food assistance program (β = 0.06, 95% CI: 0.00, 0.12, *p* = 0.046).

**Table 3:** Ordinary least squares regression between large retail store exposure to NEOP interventions and percent of total dollars spent on fruits and vegetables among survey participants exposed to at least one store visual, Los Angeles County, 2014–2015.

	Model 1 (bivariate)			Model 2 (multivariable)		
	β	95% CI	*p*-Value	β	95% CI	*p*-Value
Watched cooking demonstration in store (ref = No)	−0.01	−0.07, 0.06	0.807	−0.02	−0.07, 0.07	0.982
Report seeing at least one Champions ad in community (ref = No)	−0.01	−0.06, 0.04	0.660	0.00	−0.07, 0.03	0.480
Female (ref = Male)	0.08	0.02, 0.13	0.005	0.07	0.01, 0.13	0.029
Age (ref = 18–35)						
36–50	0.02	−0.04, 0.09	0.502	0.03	−0.04, 0.10	0.402
51–64	0.00	−0.07, 0.07	0.948	0.00	−0.08, 0.08	0.978
65+	−0.03	−0.11, 0.05	0.471	−0.05	−0.15, 0.05	0.367
Latino (ref = non-Latino)	−0.01	−0.13, 0.10	0.831	−0.05	−0.17, 0.08	0.489
Educational attainment (ref ≤ high school)						
High school	−0.01	−0.06, 0.03	0.555	−0.01	−0.07, 0.04	0.589
Associates	−0.04	−0.15, 0.07	0.457	−0.03	−0.14, 0.09	0.615
Bachelors +	−0.06	−0.17, 0.05	0.264	−0.03	−0.16, 0.09	0.611
Currently working (ref = no)	−0.04	−0.09, 0.01	0.081	−0.01	−0.10, 0.01	0.097
Enrolled SNAP/CalFresh (ref = no)	0.05	0.00, 0.10	0.049	0.06	0.00, 0.12	0.046
Enrolled WIC (ref = no)	−0.01	−0.06, 0.04	0.640	−0.04	−0.10, 0.03	0.249
Enrolled head start (ref = no)	−0.08	−0.21, 0.06	0.258	−0.14	−0.29, 0.00	0.056
Neighborhood location (ref = Downtown Store 1)						
Downtown Store 2	0.02	−0.10, 0.13	0.79	0.00	−0.12, 0.13	0.983
Downtown Store 3	0.03	−0.08, 0.13	0.63	−0.01	−0.12, 0.11	0.914
East Los Angeles Store 4	0.01	−0.09, 0.12	0.84	0.01	−0.10, 0.13	0.817
South Los Angeles Store 5	0.02	−0.08, 0.11	0.74	0.02	−0.09, 0.12	0.726
South Los Angeles Store 6	0.01	−0.09, 0.10	0.87	0.01	−0.10, 0.11	0.901
Correct knowledge of fruit and vegetable recommendation (ref = incorrect)	0.02	−0.02, 0.07	0.320	0.03	−0.02, 0.08	0.179

### 3.2 Store environment findings

Most produce sections were placed near the store entrance (4 of 6 stores) and the remaining stores' produce sections were to the right of the store, but not near the entrance (2 of 6). There was an average of 97 visuals (signs) in the produce section (i.e. store signage, shelf talkers, display cases) from multiple different sponsors. Three stores had one NEOP promotional visual, one store had 9 promotional visuals, and two stores did not have the visuals in their produce section. In 3 of the 6 stores, in addition to the produce section, fresh fruits were also found in high impact areas while vegetables were found in high impact areas in 2 of the 6 stores. Across all sections of the store, all stores had some type of healthy messaging, with themes of “encouraging more consumption of fruits and vegetables,” “WIC-qualifying produce awareness,” “nutrition facts”, and “general health promotion messages.” Healthier messages were also placed in the cereal aisle, frozen section, canned section, and dairy case. In two of the stores, the promotional visuals were found in other sections of the store including the cereal section and frozen section.

On average across the 6 stores, there were 8.8 checkout aisles, with a range of 5 to 16. Almost all the checkout aisles displayed candy (88%), more than half displayed soda (58%), and about one third displayed water (31%). There was no healthy messaging in any of the checkout aisles. All soda aisles had price promotion signage for reduced price and/or quantity discount and prompts (store signage, shelf-talkers, and display cases).

## 4 Discussion

In this sample of patrons from six large grocery stores in Los Angeles County, findings from the present study provided little evidence that seeing a NEOP Retail Program visual or watching a cooking demonstration was associated with increased fruit and vegetable purchases. While approximately 25% of participants reported seeing a store visual and 9% watched a cooking demonstration, changing shopping behaviors may be particularly difficult with low-income populations who often have restricted budgets. Prior studies have found that introducing promotional materials in a store was not associated with making produce purchases (Gittelsohn, 2010; Kristal et al., 1997; Dougherty et al., 1990). Although grocery stores may offer a variety of fresh fruits and vegetables, they also are inundated with low-cost, unhealthy food options; thus, modest interventions may be insufficient to improve health behaviors right away without complementary financial incentives or enrollment in SNAP. Research has shown that a monetary incentive, either through price discounts or coupons, seems promising in increasing purchases of healthier food options (Polacsek et al., 2018; Olstad et al., 2017, Ni Mhurchu et al., 2010, Waterlander et al., 2013;). It should be noted that two of the six stores in the study did not contain any promotional materials when the environmental scan was conducted. However, some of the participants at these stores reported they had seen the promotional materials throughout the store or elsewhere. It is likely that the materials may have been placed in the store and were then taken down before the environmental scan was conducted.

On average, the present survey sample appeared to be spending a relatively high proportion of their total grocery dollars on produce (43%). In a similar study focusing on lower-income populations in Los Angeles, Ortega et al. (2016) found that 34% of participants' total dollars were spent on fruits and vegetables at follow-up (Ortega et al., 2016). In another study, however, Steel-Adjognon and Weatherspoon found that only around 6% of the expenditures of lower-income Hispanics who frequent a supermarket in Detroit, MI, were spent on produce (Steele-Adjognon and Weatherspoon, 2017). Nationally, around 12% of grocery shopping dollars are spent on produce (Supermarket Facts, 2017). The higher than average fruit and vegetable expenditures seen in the present study may be due to social desirability bias, recall bias, or perhaps because produce may be more expensive in the communities where the study team surveyed.

Present study findings did show that certain socio-demographic groups, including females and those with higher education, purchased more fruits and vegetables, as compared to males and those with lower education. This was not a surprise and was expected since previous research has shown that women are more likely than men to consume fruits and vegetables (Blanck et al., 2008; Emanuel et al., 2012). The grocery store setting may not be the best setting to reach the male population as it has been found that females tend to be the primary shoppers (Goodman, 2008). However, there is an increasing trend towards increasing male participants in nutrition programs in the worksite setting (Taylor et al., 2013). Past research has also shown that education shares a strong association with nutrition-based diet quality and may impact the acquisition of information about healthy eating practices (Raffensperger et al., 2010; Turrell and Kavanagh, 2006).

A promising finding from this study was that participants who were enrolled in SNAP/CalFresh increased their purchases of fruits and vegetables after seeing a store visual; this was in contrast to those not enrolled in this food assistance program. The goal of SNAP-Ed is to complement and encourage SNAP enrollees and SNAP-eligible populations to improve nutrition and prevent or reduce diet-related chronic disease and/or obesity. Research has shown that this group may be more likely to consume less nutritious food (Nguyen et al., 2014; Leung et al., 2012; Gleason et al., 2000); albeit a recent study found that both SNAP and non-SNAP patrons, on average, buy similar groceries (Garasky et al., 2016). Past research has suggested that dual enrollment in SNAP and WIC is associated with increased consumption of fruits and vegetables; unfortunately, the increased access to more SNAP funds was also associated with consumption of other unhealthy foods and beverages like soda (Liu et al., 2015). Future research may be needed to better understand whether visuals can prompt SNAP recipients to purchase healthier foods or to describe how increased SNAP benefits can lead to healthier food purchases among program beneficiaries.

### 4.1 Limitations

The present study has several limitations with its design and implementation. First, the primary outcome variable, total dollars spent on fruits and vegetables, was self-reported since access to accurate sales data was not available for the sampled study period. While the study question asked participants to report dollars spent on an average week, these participants may have inaccurately reported or have experienced recall bias on this measure. Second, cooking demonstrations were not regularly conducted across all six stores, thus some participants may not have had the opportunity to participate in this particular intervention. It is plausible that if more people were exposed to this component of the large retail NEOP interventions, patrons who participated in the survey may have changed their purchasing behaviors. Third, social desirability bias may have influenced responses to the outcome question. Fourth, to measure NEOP intervention exposure, participants were shown NEOP visuals and media advertisements and asked if they had seen any visuals in the store or any of the advertisements in the community. Using aided questions could have increased recall bias. Fifth, due to staff constraints and timing, the study was not able to verify if the promotional materials were posted consistently throughout the course of the project. Finally, this study was not able to utilize a control group in its design and it did not have available pre-test data that could be incorporated during project planning. Future nutrition programs that focus on the large retail environment should incorporate these evaluation research design elements to better establish intervention exposure and how it may shape shopping behavior.

## 5 Conclusions

The present study highlights the need to broaden grocery store interventions beyond increasing individual awareness of fruits' and vegetables' importance. In particular, POP interventions may play an important role in changing purchasing behaviors among store patrons by complementing store visuals with price incentives (or other in-store measures) to purchase more produce. A more intense and robust NEOP Retail Program in Los Angeles County may have led to a greater impact on fruit and vegetable purchases if the program had focused on SNAP enrolled populations. Taken together, the NEOP store interventions may still have added educational value by increasing knowledge and influencing food choices, even if they did not *individually* increase store patron purchases of fruits and vegetables. In other words, missed opportunities remain for coupling SNAP-Ed resources so that SNAP-enrolled households can optimize their food assistance funding to purchase more produce.

## Disclosure

The authors report no financial disclosures or conflicts of interest.

## Footnotes

2 SNAP-Ed = Supplemental Nutrition Assistance Program Education

3 NEOP = Nutrition Education and Obesity Prevention

4 POP = Point-of-purchase interventions

5 DPH = Los Angeles County Department of Public Health
